# Evaluation of survivin expression in prostate specimens of patients with prostate adenocarcinoma and benign prostate hyperplasia underwent transurethral resection of the prostate or prostatectomy

**DOI:** 10.1186/s40064-016-2283-5

**Published:** 2016-05-14

**Authors:** Mansooreh Eslami, Tahere Khamechian, Tahere Mazoochi, Hasan Ehteram, Mojtaba Sehat, Javad Alizargar

**Affiliations:** Kashan University of Medical Sciences, Kashan, Islamic Republic of Iran; Anatomical Science Research Center, Kashan University of Medical Sciences, Kashan, Islamic Republic of Iran; Gametogenese Research Center, Kashan University of Medical Sciences, 5th Km Qotbe Ravandi Blv, Kashan, Islamic Republic of Iran; Student Research Committee, Kashan University of Medical Sciences, Kashan, Islamic Republic of Iran

## Abstract

**Background:**

Survivin is a newly found member of the inhibitors of apoptosis proteins, which plays a certain role in cancer. Survivin has a distinctly different expression in cancers, including prostate cancer. We are searching for the relationship between survivin levels in normal prostate tissue, benign prostate hyperplasia (BPH), and prostate adenocarcinoma in this study.

**Results:**

The study surveyed 282 prostate samples, 94 normal, 94 BPH, and 94 prostate adenocarcinoma samples. Survivin expression was absent in normal prostate tissues. In the BPH group, the survivin expression level was higher than that of the normal group. In the adenocarcinoma group, the survivin expression level was higher than that of the BPH group. There was a significant association between survivin expression level and the adenocarcinoma stage.

**Conclusion:**

Although there is no expression of survivin in normal prostate tissue, its expression is slightly positive in BPH. High survivin expression is related to a higher Gleason score in the adenocarcinoma of the prostate.

## Background

The inhibitors of apoptosis (IAP) proteins are a group of proteins that mediate several pathways, including cell cycle, immunity, inflammation, and cell death (de Almagro and Vucic 2012). One of the recently discovered IAP proteins is survivin. It is the smallest member in the IAP family and is mainly expressed in the fetal tissue but has a known role in many cancers, such as bladder cancer, non-small cell lung cancer, esophageal cancer, breast carcinoma, gastric carcinoma, recurrent colorectal carcinoma, rectal cancer, neuroblastoma, and prostate cancer (Ambrosini et al. 1997; Kishi et al. 2004). Survivin is somehow unique in the IAP family, because of its structure and its definite difference of expression in cancer, including prostate cancer tissue as compared the normal tissue. Survivin is also associated with cancer prognosis and progression (Zhang et al. 2009). The absence of survivin in normal tissues and its over expression in tumors, and the correlation of survivin and poor prognosis of cancer, attracts many researchers to evaluate its potential therapeutic and diagnostic values (Lladser et al. 2011).

Since 1966, Gleason Grading is used in evaluation prostate cancer and it is the strongest prognostic factor in prostate cancer (Gleason 1966; Sauter et al. 2016). Its strong feature is that it is not influenced by cellular morphology and is based on the cancer features. Although tumor volume and PSA level increase may trigger treatment, the main treatment criteria is a Gleason score of 7 or more (Komisarenko et al. 2016). The Gleason grading system was updated in 2005 at a consensus conference of international experts in urological pathology (Epstein 2010) and they agreed that Gleason score between 2 and 4 should not be given on prostate biopsies. This update is currently recommended in international guidelines.

Studies have shown that the level of survivin expression gradually increases from normal prostate tissue and low risk prostate cancer to high risk prostate cancer and is highest in metastatic prostate cancer (Shariat et al. 2004). Some studies show that survivin has a higher expression in benign prostatic hypertrophy (BPH) and that it correlates with BPH parameters (Shariat et al. 2005). However, there are also some studies that confirm that the level of survivin in BPH is not higher than that in normal prostatic tissue (Yu et al. 2010). Although few studies have been conducted to clarify the relationship between survivin and the grading of prostate cancer, this relation appears contradictory. If this relationship is definite, we can use survivin as a prognostic factor and therapeutic target. In this study, we are searching for the relationship between survivin level in normal prostate tissue, BPH, and prostate adenocarcinoma.

## Methods

The study sample size was calculated on the basis of the survivin level in BPH and the adenocarcinoma of the prostate in a previous study by Rodríguez-Berriguete et al. (Rodríguez-Berriguete et al. 2010). We assumed that there is no survivin expression in normal prostate samples. According to the 95 % confidence interval and power of 20 %, the sample size was calculated as 94 in each group (normal prostate, BPH, and adenocarcinoma of the prostate).

Samples obtained using prostatectomy or TURP and adenocarcinoma and BPH samples diagnosed on the basis of H&E staining. Samples from biopsies were not included in our study, because of the small size of the samples, that could negatively affect the accuracy of staining and Gleason grading. Normal prostate samples obtained from normal glands adjacent to adenocarcinoma or BPH tissue, are consistent with the method employed by Shariat et al. (Shariat et al. 2004).

These samples were obtained from patients admitted to Shahid Beheshty Hospital between 2002 and 2014. Normal prostate and BPH sampling was based on a table of random numbers from the patients admitted to the same hospital during the same period. All prostate samples archived in the pathology ward which had no history of urinary tract infection and prostatitis were included in the study. All of the cases with incomplete and damaged data, with improper staining, and results obtained via needle biopsy were excluded. There are some reports that suggest inflammation as a trigger of increased survivin expression in prostate cells (St. Sauver and Jacobsen 2008), therefore, samples with infiltration of inflammatory cells were also excluded from our study.

Samples were obtained with the usual method of sampling (Dabbs 2013): 5-µm paraffinated blocked sections were obtained from all prostate samples. HaematoxylinEosine (H&E) and Immunohistochemistry (IHC) methods used after sample preparation: (1) Samples were subjected to 60 °C for 2 h. (2) Rehydration was conducted with xylolose, alcohol, and distilled water for 15 min, 10 min, and 5 min, respectively. (3) Endogenous peroxidase activity removal: samples were put in 3 % hydrogen peroxidase for 20 min and washed with phosphate-buffered saline twice, for 5 min each time.

(4) Antigen retrieval: Samples are paled in a citrate puffer (pH 6) and in the autoclave of 134° and 1.5–2 bar pressure for 10 min. They are then let to cool at room temperature for 20 min and wash with distilled water thereafter. (5) Primary survivin antibody is added to the samples for 1 h. (6) They are washed with phosphate-buffered saline (PBS) three times for 5 min each time. (7) Secondary antibody is added and placed for 30 min at room temperature. (8) PBS washing. (9) 3,3′-diaminobenzidine (DAB) chromogen: samples are placed in the diluted solution for 10 min (1.9 DAB with DAB substance ratio). (10) Hematoxylin staining of the background for 1 min, followed by washing. (11) Dehydration of the slides with 96 % alcohol and 100 % xylol, every two sides, 10 min each side. (12) Mounting of the slides in preparation for viewing under the light microscope.

**Fig. 1:**
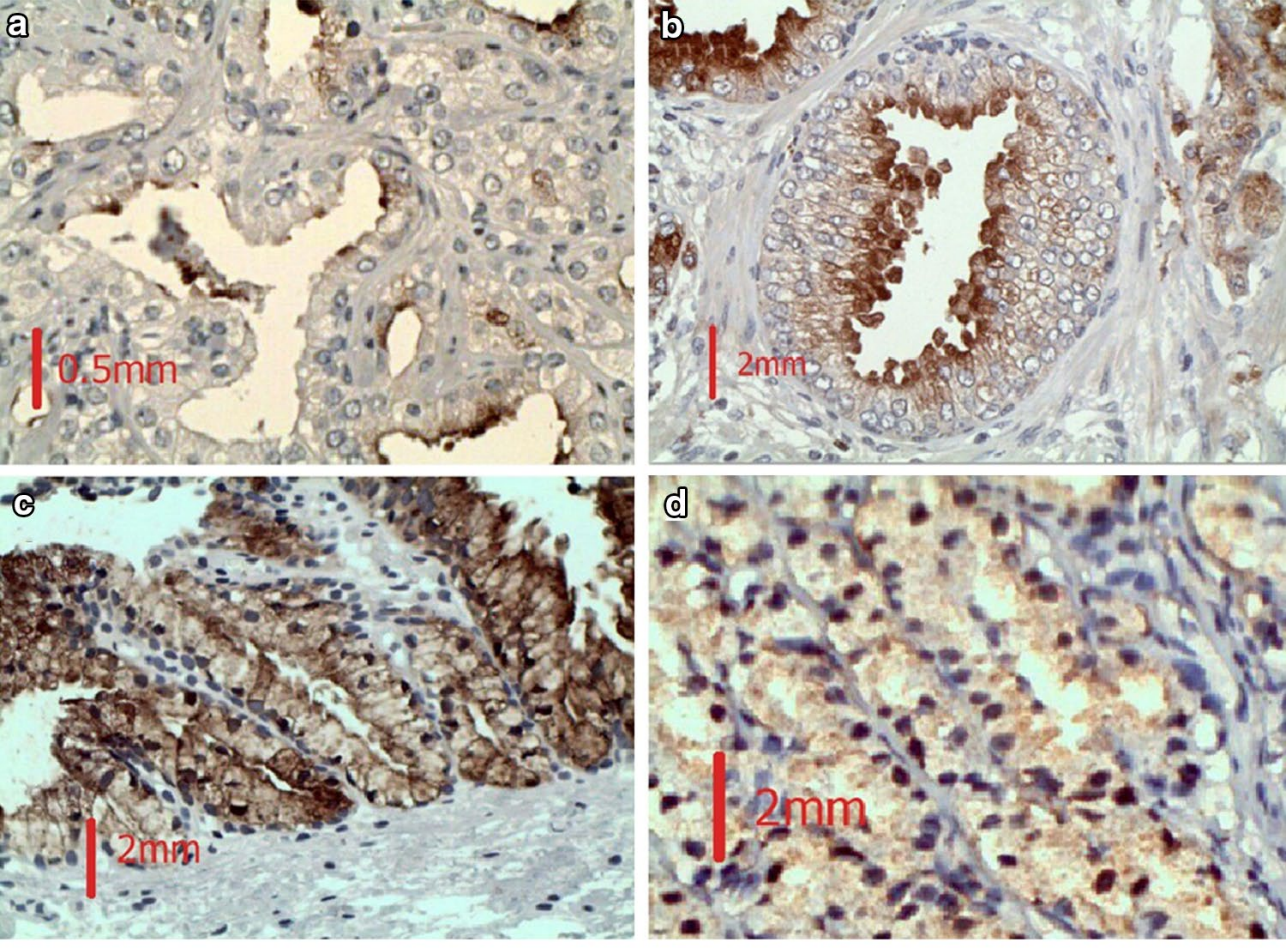
Different levels of survivin staining in the samples. **a** Negative, <10 % of epithelial cells weakly stained, **b** 1+, 10–29 % of epithelial cells stained weakly to moderate, **c** 2+, 30–49 % of epithelial cells stained moderately to intensive, **d** 3+, more than 50 % of epithelial cells stained intensely

Histopathological diagnosis of the H&E stained slides are determined and the parts with no pathological findings are considered normal (Fig. 1). Normal prostate tissue is made of separate, small, regular glands with one epithelial and one basal cell layer. We can see a glandular hyperplasia, cystic and dilated glands, multi-layer epithelium or papillary formation in BPH. In the adenocarcinoma slides, diagnosis was based upon the Gleason criteria. Final grading is the sum of primary (dominant appearance of the tumor) and secondary (other sites of the tumor) survivin grading. IHC evaluation of the stained slides comprised two groups of positive and negative. Positive samples were categorized as +1, +2, and +3 groups. No staining or weak staining of up to 10 % of the cells was considered negative. Weak to moderate staining in 10–29 % of the cells was considered +1. Moderate to intense staining in 30–49 % of the cells was considered 2+. Intense staining in more than 50 % of the cells was considered 3+. Intensity of staining was evaluated by two pathologists. If they did not categorize a sample similarly, the third pathologist categorized the sample. According to the guidelines on the antibody kit, bladder transitional carcinoma cell was considered a positive control and normal cervix tissue a negative control. H&E and IHC staining were synchronously and similarly conducted on all the samples and on the positive and negative control simultaneously.

All collected data, including histopathological diagnosis, tumor grade, IHC results (positive or negative), and staining intensity were recorded, along with the patients’ age and serum PSA level.

### Statistical analysis

All collected data were analyzed using SPSS version 17. We described survivin and other variables according to their distribution. We compared the survivin level between groups using the Chi squared test. Logistic regression analysis was used to remove the confounding factors. p was considered significant at the level of 0.05.

## Results

This study surveyed 282 prostate samples: 94 normal, 94 BPH, and 94 prostate adenocarcinoma samples. The patients’ mean (SD) age in the normal, BPH, and adenocarcinoma groups were 73.7 (8.9), 72.4 (7.6), and 75.6 (7.9) years, respectively. Demographic data of each group can be found in Table 1.

**Table 1:** Demographic data of cases in different histopathological groups

Group	Normal	BPH	Adenocarcinoma
Age (mean ± SD)	74 ± 9	72 ± 8	76 ± 8
Age range	50–95	50–89	52–95
Total number	94	94	94

**Fig. 2:**
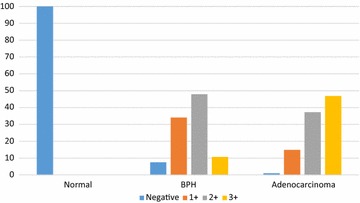
Survivin expression percentage levels in different histopathological groups of prostate samples

All samples in the normal prostate group were stained less than 10 % for survivin and considered negative. In the BPH group, 7 samples (7.4 %) were negative, 32 samples (34 %) were 1+, 45 (47.9 %) were 2+, and 10 (10 %) were 3+. In the adenocarcinoma group, 1 sample (1.1 %) was negative, 16 (14.9 %) were 1+, 35 (37.2 %) were 2+, and 44 (46.8 %) were 3+ for survivin (Fig. 1). Figure 2 shows different levels of survivin staining. The variation of survivin expression between these three groups was considered meaningful, thus there is a statistical difference between survivin expression in normal, BPH and adenocarcinoma (p < 0.01) (Table 2).

**Table 2:** Survivin expression levels in different histopathological groups of prostate samples

Histology	Survivin level (%)					p value*
	Negative	1+	2+	3+	Total	
Normal	94 (100)	0	0	0	94 (100)	<0.001
BPH	7 (7.4)	32 (34)	45 (47.9)	10 (10.6)	94 (100)	
Adenocarcinoma	1 (1.1)	14 (14.9)	35 (37.2)	44 (46.8)	94 (100)	
Total	102 (36.2)	46 (16.3)	80 (28.4)	54 (19.1)	282 (100)	

* Pearson Chi Square test

These three groups were compared separately. It was found that survivin levels in the adenocarcinoma group were considerably greater than those in the normal group. Comparing the survivin levels of the BPH and adenocarcinoma groups with the normal group revealed a significant difference (p < 0.001).

The risk of adenocarcinoma, comparing BPH group, is increased 3.1 times (95 % CI: 2.0, 4.8) with every level of survivin increasing and after adjusting according to age, this relation stayed unchanged. Adenocarcinoma samples were graded according to the Gleason staging criteria. There were 19 samples (20.2 %) in the grade 7 and it was the most prevalent grade among all grades. All the seven samples in grade 10 were 3+ stained for survivin expression. In the adenocarcinoma samples, there was only one sample with negative staining for survivin. The Gleason grade of this patient was four (Table 3).

**Table 3:** Gleason staging of adenocarcinoma and survivin expression

Gleason stage	Survivin expression (%)				
	Negative	1+	2+	3+	Total
2	0	4 (57.1)	2 (28.6)	1 (14.3)	7 (7.4)
3	0	3 (42.9)	2 (28.6)	2 (28.6)	7 (7.4)
4	1 (10)	0	7 (70)	2 (20)	10 (10.6)
5	0	3 (27.3)	4 (36.4)	4 (36.4)	11 (11.7)
6	0	1 (9.1)	4 (36.4)	6 (54.5)	11 (11.7)
7	0	2 (10.5)	9 (47.4)	8 (42.1)	19 (20.2)
8	0	1 (6.7)	3 (20)	11 (73.3)	15 (16)
9	0	0	4 (57.1)	3 (42.9)	7 (7.4)
10	0	0	0	7 (100)	7 (7.4)
Total	1 (1.1)	14 (14.9)	35 (37.2)	44 (46.8)	94 (100)

The higher the Gleason grade, the greater the degree of survivin expression staining. Most (60.4 %) of the adenocarcinoma samples with Gleason grade of ≥7 were 3+ positive for survivin. The chance of a Gleason grade of ≥7 in the patients with 3+ staining was 3.15 times in patients with staining <50 % (Tables 4, 5).

**Table 4:** Comparing survivin expression and Gleason grading of >7

Gleason stage	Survivin		
	Less than 3+ (%)	3+ (%)	Total
<7	31 (62)	15 (34.1)	46 (48.9)
≥7	19 (38)	29 (65.9)	48 (51.1)
Total	50 (100)	44 (100)	94 (100)

**Table 5:** Comparing the Gleason grading of >7 with survivin expression

Survivin	Gleason grade			Odds Ratio (95 % CI)	p value
	<7	≥7	Total		
<3+	31 (67.4)	19 (39.6)	50 (53.2)	3.15 (1.35–7.35)	0.008
3+	15 (32.6)	29 (60.4)	44 (46.8)		
Total	46 (100)	48 (100)	94 (100)		

Survivin expression up to 30 % (negative and 1+ degree in our staging) is associated with a slight increase in adenocarcinoma staging; however, this association is more apparent in the upper percentages of survivin expression. This correlation is considerable overall.

The relation of the PSA level and survivin expression was statistically significant (p < 0.001) (Table 6). Most of the samples with a PSA of >10 (42.3 %) had a 3 + survivin expression, but the samples with a PSA of <4 only had 5.5 % of 3+ survivin expression. Age did not exert a considerable effect on survivin expression in BPH and adenocarcinoma groups (p > 0.05).

**Table 6:** Serum PSA level and survivin expression correlation

PSA	Survivin level					p*
	Negative	1+	2+	3+	Total	
<4	26 (28.6)	27 (29.7)	33 (36.3)	5 (5.5)	91	<0.001
4–9.9	57 (41)	17 (12.2)	38 (27.3)	27 (19.4)	139	
≥10	19 (36.5)	2 (3.8)	9 (17.3)	22 (42.3)	52	
Total	102 (36.2)	46 (16.3)	80 (28.4)	54 (19.1)	282	

* Pearson Chi square test

## Discussion

This study evaluates the expression of survivin protein in prostate samples with the IHC method. According to our results, the expression of survivin was negative in the nucleus of normal prostate samples cells, slightly positive in the BPH sample cells, and associated with Gleason staging in the adenocarcinoma group. There is a significant correlation between nuclear and cytoplasmic survivin staining (Zhang et al. 2009), which is comparable with the results of studies evaluating the cytoplasmic and nuclear survivin levels.

Some studies used normal prostate tissues biopsies (Rodríguez-Berriguete et al. 2010). Normal autopsies could not be used in this study, however, owing to methodological limitations. We could not obtain autopsies because the families did not consent.

The results of absence of survivin expression in normal prostate tissues are consistent with other studies (Shariat et al. 2004; Rodríguez-Berriguete et al. 2010). There was a similar negative expression of survivin in these studies in the normal epithelial prostate glands.

In our study, in prostate adenocarcinoma, the survivin expression was higher than BPH and this result is in accordance with the results of Rodríguez-Berriguete et al. (Rodríguez-Berriguete et al. 2010), but the study by Yu et al. (2010) suggests that survivin expression in BPH does not differ significantly from normal prostate tissue. However according to our study we cannot efficiently use 3+ surviving staining for differentiating adenocarcinoma and BPH samples. Using other markers such as alpha-methylacyl CoA racemase and myoepithelial markers such as 34βE12 and P63 appears more functional to differentiate between these two anomalies (Carswell et al. 2006; Kalantari et al. 2014). Survivin is expressed in other tumor types (Shintani et al. 2013; Fukuda and Pelus 2006), and thus, cannot be used in locating the tumor’s origin in metastasis with an unknown origin.

The results of this study showed that malignant prostate carcinoma (based on the Gleason criteria) and survivin expression co-occur, as Shariat et al. suggested. However, our results are not in accordance with those of Rodríguez-Berriguete et al. (Rodríguez-Berriguete et al. 2010). Most patients (60.41 %) with a Gleason score of ≥7 were positive for survivin with a 3+ scale; thus, high survivin expression is related to a higher Gleason score. We found the chance of a Gleason grade of ≥7 in the patients with 3+ staining was 3.15 times in patients with staining <50 %. Although, we excluded the cases with UTI and inflammation to lower the confounding factors, however, the cases were not screened for other malignancies, which may in part, increase the survivin expression. Moreover, the effects of age has been considered, and after adjusting according to age, the results remained unchanged.

We used the IHC grading developed by Shariat et al. (Shariat et al. 2004). Other computer analytical and manual methods also exist (Shinde et al. 2014). For example Dabbs et al. (Dabbs 2013) uses the Histochemical score (H score) for hormonal receptors in breast tumors and counts the non-stained nucleases. This score ranges from 0 to 300. This method was used by Mazieres et al. for the expression of EGFR in lung tumors with IHC (Mazières et al. 2013).

Besides we can use survivin as a prognostic factor, there are therapeutic advantages of measuring it. Survivin can show chemotherapeutic resistance to cisplatin, bortezomib, vincristine, tamoxifen, TNF-a and TRAIL in tumor cells (Cheung et al. 2010; Liu et al. 2010; Zhang et al. 2006; Ling et al. 2010). There is also reports that survivin suppresses radiation-induced apoptosis (Soleimanpour and Babaei 2015), so, targeting survivin can increase the sensitivity to radiotherapy in cancer patients.

The retrospective nature of this study can be counted as a limitation, and a prospective study with other grading methods would further clarify the subject. We could not screen the patients for other tumor types as survivin has been suggested as a progression factor in some cancers, including bladder (Zhang et al. 2016), colon (Li et al. 2016) and breast (Liu et al. 2016). Prospective studies can lessen these confounding factors. Although studies on prostate cancer can reveal the prognostic and therapeutic importance of survivin, further studies on survivin level in different cancers would make a valuable therapeutic and prognostic target for treating patients with other cancers, as well.

## Conclusion

We may conclude that there is no expression of survivin in normal prostate tissue but that its expression is slightly positive in the BPH and it is associated with Gleason staging. High survivin expression is related to a higher Gleason score in the adenocarcinoma of the prostate. Survivin detection with the IHC method is unhelpful for differentiating adenocarcinoma of the prostate and BPH.

## References

[CR1] Ambrosini G, Adida C, Altieri DC (1997). A novel antiapoptosis gene, survivin, expressed in cancer and lymphoma. Nat Med.

[CR2] Carswell BM, Woda BA, Wang X, Li C, Dresser K, Jiang Z (2006). Detection of prostate cancer by alpha-methylacyl CoA racemase (P504S) in needle biopsy specimens previously reported as negative for malignancy. Histopathology.

[CR3] Cheung CH, Sun X, Kanwar JR (2010). A cell-permeable dominant-negative survivin protein induces apoptosis and sensitizes prostate cancer cells to TNF alpha therapy. Cancer Cell Int.

[CR4] Dabbs DJ (2013). Diagnostic immunohistochemistry.

[CR5] de Almagro MC, Vucic D (2012). The inhibitor of apoptosis (IAP) proteins are critical regulators of signaling pathways and targets for anti-cancer therapy. Experimental oncology.

[CR6] Epstein JI (2010). An update of the Gleason grading system. J Urol.

[CR7] Fukuda S, Pelus LM (2006). Survivin, a cancer target with an emerging role in normal adult tissues. Mol Cancer Ther.

[CR8] Gleason DF (1966). Classification of prostatic carcinomas. Cancer Chemother Rep.

[CR9] Kalantari MR, Anvari K, Jabbari H, VarshoeeTabrizi F (2014). p63 is more sensitive and specific than 34βE12 to differentiate adenocarcinoma of prostate from cancer mimickers. Iran J Basic Med Sci..

[CR10] Kishi H, Igawa M, Kikuno N, Yoshino T, Urakami S, Shiina H (2004). Expression of the survivin gene in prostate cancer: correlation with clinicopathological characteristics, proliferative activity and apoptosis. J Urol.

[CR11] Komisarenko M, Wong LM, Richard PO, Timilshina N, Toi A, Evans A, Zlotta A, Kulkarni G, Hamilton R, Fleshner N, Finelli A (2016). An increase in Gleason 6 tumor volume while on active surveillance portends a greater risk of grade reclassification with further followup. J Urol.

[CR12] Li WL, Lee MR, Cho MY (2016). The small molecule survivin inhibitor YM155 may be an effective treatment modality for colon cancer through increasing apoptosis. Biochem Biophys Res Commun.

[CR13] Ling X, Calinski D, Chanan-Khan AA, Zhou M, Li F (2010). Cancer cell sensitivity to bortezomib is associated with survivin expression and p53 status but not cancer cell types. J Exp Clin Cancer Res.

[CR14] Liu JL, Wang Y, Jiang J (2010). Inhibition of survivin expression and mechanisms of reversing drug-resistance of human lung adenocarcinoma cells by siRNA. Chin Med J.

[CR15] Liu S, Huang W, Jin MJ, Fan B, Xia GM, Gao ZG (2016). Inhibition of murine breast cancer growth and metastasis by survivin-targeted siRNA using disulfide cross-linked linear PEI. Eur J Pharm Sci.

[CR16] Lladser A, Sanhueza C, Kiessling R, Quest AFG (2011). Is survivin the potential Achilles heel of cancer?. Adv Cancer Res.

[CR17] Mazières J, Brugger W, Cappuzzo F, Middel P, Frosch A, Bara I, Klingelschmitt G, Klughammer B (2013). Evaluation of EGFR protein expression by immunohistochemistry using H-score and the magnification rule: re-analysis of the SATURN study. Lung Cancer.

[CR18] Rodríguez-Berriguete G, Fraile B, de Bethencourt FR (2010). Role of IAPs in prostate cancer progression: immunohistochemical study in normal and pathological (benign hyperplastic, prostaticintraepithelialneoplasia and cancer) human prostate. BMC Cancer.

[CR19] Sauter G, Steurer S, Clauditz TS, Krech T, Wittmer C, Lutz F, Lennartz M, Janssen T, Hakimi N, Simon R, von Petersdorff-Campen M, Jacobsen F, von Loga K, Wilczak W, Minner S, Tsourlakis MC, Chirico V, Haese A, Heinzer H, Beyer B, Graefen M, Michl U, Salomon G, Steuber T, Budäus LH, Hekeler E, Malsy-Mink J, Kutzera S, Fraune C, Göbel C, Huland H, Schlomm T (2016). Clinical utility of quantitative Gleason grading in prostate biopsies and prostatectomy specimens. Eur Urol.

[CR20] Shariat SF, Lotan Y, Saboorian H, Khoddami SM, Roehrborn CG, Slawin KM, Ashfaq R (2004). Survivin expression is associated with features of biologically aggressive prostate carcinoma. Pubmed Cancer.

[CR21] Shariat SF, Ashfaq R, Roehrborn CG, Slawin KM, Lotan Y (2005). Expression of survivin and apoptotic biomarkers in benign prostatic hyperplasia. J Urol.

[CR22] Shinde V, Burke KE, Chakravarty A, Fleming M, McDonald AA, Berger A (2014). Applications of pathology-assisted image analysis of immunohistochemistry-based biomarkers in oncology. Vet Pathol.

[CR23] Shintani M, Sangawa A, Yamao N, Kamoshida S (2013). Immunohistochemical expression of nuclear and cytoplasmic survivin in gastrointestinal carcinoma. Int J ClinExpPathol.

[CR24] Soleimanpour E, Babaei E (2015). Survivin as a potential target for cancer therapy. Asian Pac J Cancer Prev.

[CR25] St. Sauver JL, Jacobsen SJ (2008). Inflammatory mechanisms associated with prostatic inflammation and lower urinary tract symptoms. Curr Prostate Rep.

[CR26] Yu HB, Han XB, Liang YQ, Liu JG, Wang H (2010). Expression of osteopontin and survivin in prostate cancer and the clinical significance. Southern Med Univ.

[CR27] Zhang MC, Hu CP, Chen Q (2006). Effect of down-regulation of survivin gene on apoptosis and cisplatin resistance in cisplatin resistant human lung adenocarcinoma A549/CDDP cells. Zhonghua Zhong Liu Za Zhi [Chin J Oncol].

[CR28] Zhang M, Ho A, Hammond EH, Suzuki Y, Bermudez RS, Lee RJ, Pilepich M, Shipley WU, Sandler H, Khor LY, Pollack A, Chakravarti A (2009). Prognostic value of survivin in locally advanced prostate cancer: study based on RTOG 8610. Int J Radiat Oncol Biol Phys.

[CR29] Zhang J, Wang S, Han F, Li J, Yu L, Zhou P, Chen Z, Xue S, Dai C, Li Q (2016). MicroRNA-542-3p suppresses cellular proliferation of bladder cancer cells through post-transcriptionally regulating survivin. Gene.

